# Transcriptome and Degradome of microRNAs and Their Targets in Response to Drought Stress in the Plants of a Diploid and Its Autotetraploid *Paulownia australis*

**DOI:** 10.1371/journal.pone.0158750

**Published:** 2016-07-07

**Authors:** Suyan Niu, Yuanlong Wang, Zhenli Zhao, Minjie Deng, Lin Cao, Lu Yang, Guoqiang Fan

**Affiliations:** 1 Institute of Paulownia, Henan Agricultural University, 95 Wenhua Road, Jinsui District, 450002, Zhengzhou, Henan, P.R. China; 2 College of Forestry, Henan Agricultural University, 95 Wenhua Road, Jinsui District, 450002, Zhengzhou, Henan, P.R. China; Nanjing Agricultural University, CHINA

## Abstract

MicroRNAs (miRNAs) are small, non-coding RNAs that play vital roles in plant growth, development, and stress response. Increasing numbers of studies aimed at discovering miRNAs and analyzing their functions in plants are being reported. In this study, we investigated the effect of drought stress on the expression of miRNAs and their targets in plants of a diploid and derived autotetraploid *Paulownia australis*. Four small RNA (sRNA) libraries and four degradome libraries were constructed from diploid and autotetraploid *P*. *australis* plants treated with either 75% or 25% relative soil water content. A total of 33 conserved and 104 novel miRNAs (processing precision value > 0.1) were identified, and 125 target genes were identified for 36 of the miRNAs by using the degradome sequencing. Among the identified miRNAs, 54 and 68 were differentially expressed in diploid and autotetraploid plants under drought stress (25% relative soil water content), respectively. The expressions of miRNAs and target genes were also validated by quantitative real-time PCR. The results showed that the relative expression trends of the randomly selected miRNAs were similar to the trends predicted by Illumina sequencing. And the correlations between miRNAs and their target genes were also analyzed. Furthermore, the functional analysis showed that most of these miRNAs and target genes were associated with plant development and environmental stress response. This study provided molecular evidence for the possible involvement of certain miRNAs in the drought response and/or tolerance in *P*. *australis*, and certain level of differential expression between diploid and autotetraploid plants.

## Introduction

Plants are always adversely affected by variety of biotic and abiotic factors. Drought is one of the major external factors that threaten plant development, growth, and production [1]. When under drought stress, many plants evolve special mechanisms to adapt to such stress via physiological response and molecular changes such as modification of metabolic processes, gene expression, and signal transduction pathways [2]. Understanding the mechanisms involved in plants’ responses to water deficit will provide a molecular basis for improving plants’ tolerance to drought.

*Paulownia australis* is a fast-growing deciduous hardwood species, widely distributed in China where it is cultivated on farmland for forestation, the biofuel production, paper and furniture manufacturing for its good characteristics, such as rapid growth, high ignition point, rot resistance, straight grain, knot-free wood with a satiny luster, and high biomass production [3, 4]. Polyploidy plays an important role in the speciation and evolution of plants [5] and sometimes induce better resistance under various stresses compared with their corresponding diploid progenitors [6, 7]. To obtain potential benefits from polyploidy and enlarge the excellent germplasm of *P*. *australis* (2n = 40), autotetraploid *P*. *australis* plants (4n = 80) were generated from diploid plants by using *in vitro* treatment with colchicine in 2009 [8]. Soon after that, the physiological responses of autotetraploid and diploid Paulownia plants to drought stress tolerance were studied using the 25% and 75% relative soil water contents [9]. During drought stress, the leaves of both diploid and autotetraploid plants decreased in water and chlorophyll contents but increased in superoxide dismutase (SOD) activity, soluble protein content, relative conductivity, soluble sugar, proline contents and malondialdehyde (MDA) contents [9]. However, soluble sugar, soluble protein and proline contents are higher in the autotetraploid plants than that in the diploid plants under drought stresses [9]. Ploidy variation is known to provide a type of variation in gene expression [10]. There is epigenetic interaction between redundant genes [11]. Although transcriptome analysis identified several drought-responsive genes in *Paulownia australis* plants [12], the expression patterns of miRNAs in diploid and autotetraploid Paulownia under the drought conditions are largely unknown.

MicroRNAs (miRNAs) are 18–25 nt long, single-stranded, non-coding RNAs derived from hairpin stem-loop type precursors (pre-miRNAs). MiRNAs regulate gene expression through mRNA cleavage, translational repression, or DNA methylation [13–15]. Double-stranded miRNAs (miRNA/miRNA*) are obtained by cutting the stem-loop region of primary transcripts [16, 17]. Then, the miRNA* strand is degraded and the miRNA strand binds to the RNA-induced silencing complex (RISC), which includes endonuclease argonaute (AGO) proteins that are responsible for target mRNA degradation [18]. In plants, miRNAs play vital roles in diverse regulatory pathways and are involved in almost all the developmental processes, including leaf development [19], floral development [20], stem development [21], and root development [22]. MiRNAs involved in response to drought stress have been identified in many plants, including *Arabidopsis* [23–25], creeping bentgrass (*Agrostis stolonifera*) [26, 27] rice [28, 29], soya bean [30], cotton [31], and Populus [32]. The abscisic acid (ABA) is a critical regulator for plant adaptation various biotic and abiotic stresses, and also involved in control of a wide range of physiological processes, such as seed germination and plant growth [25, 33, 34]. It had been reported that ABA could induce tolerance to drought stress in plants. Recently, an increasing number of studies have demonstrated that several drought stress response miRNAs through regulating their target genes that are associated with the ABA-dependent and ABA-independent pathways to adapt to water deficit. For example, in *Arabidopsis*, miR169 was reported to play a vital role in the plant’s drought stress response; miR169 was down-regulated through an ABA-dependent pathway while its target gene *NFYA5*, which encodes a nuclear transcription factor, was up-regulated [23]. On the contrary, another miRNA, miR394, positively regulates Arabidopsis plant tolerance to drought stress in an ABA-dependent manner, as miR394-overexpressing plants displayed higher tolerance to drought than wild control plants [25]. Li et al. had demonstrated that the miR168a-overexpressing *Arabidopsis* plants or AGO1 (ago1-27) loss-of-function mutants showed ABA hypersensitivity and drought tolerance, while mir168a-2 mutants displayed ABA hyposensitivity and drought hypersensitivity [35]. Osa-miR319a-overexpressing transgenic *creeping bentgrass* plants exhibited morphological changes and enhanced salt and drought tolerance via downregulated its target genes encoding the TCP transcription factors [26, 27]. In rice, the transport inhibitor response 1 gene (*TIR1*), which was targeted by miR393, was up-regulated under drought stress, causing the attenuation of plant growth and development [28]. In cotton, some miRNAs and their predicted targets were found to be differentially expressed under drought condition, including (miR159; teosinte branched 1-cycloidea-PCF transcription factor 3), (miR395; ATP sulfurylase 1), and (miR396; growth regulating factor 1) [31]. Together, these finding suggest that miRNAs play important roles in the drought stress response in plants.

Recently, miRNAs in the Paulownia species have been subsequently reported. Several miRNAs and their target genes associated with chromosome doubling and in response to Paulownia witches’ broom phytoplasma in Paulownia species have been identified [36–41]. Nonetheless, miRNAs involved in the drought response have not been reported in *P*. *australis* species. Thus, understanding the functions of vital microRNAs (miRNAs) and their target genes may help clarify the mechanisms involved in the drought stress response in Paulownia plants. This present study aims to gain insights into the role of *Paulownia* miRNAs under drought stress, the sequencing libraries were constructed from the diploid and autotetraploid *P*. *australis* that have been treated with either 75% or 25% relative soil water content. The differentially expressed miRNAs in diploid and autotetraploid *P*. *australis* under drought stress were identified and their targets were obtained by degradome sequencing.

## Materials and Methods

### Plant material and drought treatments

An autotetraploid clone of *Paulownia australis* was obtained by *in vitro* treatment of a diploid *P*. *australis* clone with colchicine previously [8]. This diploid and its derived autotetraploid clone were used in the present study. They were the same genome with the difference at the ploidy level. The 30-day old *in vitro* subcultured plantlets of diploid and autotetraploid *P*. *australis* were transferred into garden soil. After 30 days, the plants were transferred into larger nutrition pots (20 cm in diameter at the bottom and 20 cm deep) with the same garden soil. After 50 days, 18 diploid plants and 18 autotetraploid plants (the same developmental period within each ploidy group) under the same growth conditions were subjected to drought conditions in a water controlling experiment according to the method of Zhang et al. [9]. At least three biological duplicates were prepared for each condition. Diploid and autotetraploid *P*. *australis* plants were treated with 75% relative soil water content (PA2 and PA4) as the control samples, and with 25% relative soil water content (PA2H and PA4H) as the drought-stressed samples. Three individuals from each sample (PA2, PA2H, PA4, and PA4H) were chosen and their leaves were harvested 12 days later, immediately frozen in liquid nitrogen, and stored at −80°C for further used to construct the small RNA and degradome libraries.

### Total RNA extraction and small RNA sequencing

Total RNA was extracted from the leaves of each group using Trizol reagent (Invitrogen, Carlsbad, CA, USA) according to the manufacturer’s instructions. Small RNA (sRNA) libraries were built and sequenced on an Illumina GAIIx platform (Illumina, San Diego, CA, USA). Briefly, sRNA fragments from 18 to 30 nt long were separated and purified by polyacrylamide gel electrophoresis, then ligated to 5′ and 3′ adaptors using T4 RNA ligase (Takara, Dalian, China). The adaptor-ligated sRNAs were subsequently transcribed to single-stranded cDNA and amplified by 12 cycles of PCR. The products were used to build the four libraries (PA2, PA2H, PA4, and PA4H) for Illumina sequencing. The data used in the study have been deposited in the NIH Short Read Archive database (http://www.ncbi.nlm.nih.gov/sra) under the Accession number SRP067530 (PRJNA306359).

### Bioinformatics analysis and identification of miRNAs

After removing low quality reads, adapters, and contaminated reads from the sequencing data, the clean reads were obtained. The length distribution of the clean reads was determined and the reads were mapped onto the sequences in the *Paulownia* UniGene Database (http://www.ncbi.nlm.nih.gov/sra; SRP031770). Sequences that matched non-coding RNAs, namely rRNAs, tRNAs, snRNAs, and snoRNAs were removed. The remaining unique sequences were aligned with known miRNAs in miRBase 21.0 with a maximum of two mismatches allowed. Mireap (https://sourceforge.net/projects/mireap/) and RNAfold (http://rna.tbi.univie.ac.at/cgi-bin/RNAfold.cgi) were used to predict novel miRNAs from the remaining sRNAs that did not match known miRNA sequences. Additionally, the minimal folding free energy (MFE, DG kcal/mol), adjust minimal folding free energy (AMFE), and the minimal folding free energy index (MFEI) were calculated according to the previous report [42, 43]. The basic criteria [44] were used to screen potential novel miRNAs as follow: 1) sequences could fold secondary structures; 2) the mature miRNA and miRNA* were present in the opposite stem-arms of the hairpin precursors with two nucleotide, 3′ overhangs; 3) predicted secondary structure had the lower minimal folding free energy (< = -18 kcal/mol); 4) the number of mature miRNA had no fewer than 5 in the alignment. Furthermore, to authenticate the potential novel miRNAs, the processing precision rates of the secondary structures were calculated, and the miRNAs with the precision values < 0.1 were removed [45, 46].

### Differential expression analysis of conserved and novel miRNAs

Each miRNA was normalized to the total clean miRNA reads in each library * 1 million. (Normalized expression = actual miRNA count/total count of clean reads × 1,000,000). Fold changes in gene expression of each miRNA in the treatment library were calculated relative to that of the control library or between any two libraries (fold change = log2 (miRNA normalized read counts in one library/miRNA normalized read counts in another library)). MiRNAs with fold changes > 1 or < −1 and with *P* ≤ 0.05 were considered to be up-regulated or down-regulated, respectively, in response to drought stress. The *P*-value was calculated according to previously established methods [47].

$$P\left(x|y\right)=(\frac{N_{2}}{N_{1}})\frac{\left(x+y\right)!}{x!y!{\left(1+\frac{N_{2}}{N_{1}}\right)}^{\left(x+y+1\right)}}$$

$$C\left(y\leq y_{\min}|x\right)=\sum_{y=0}^{y\leq y_{\min}}\text{p}(y|x)$$

$$D\left(y\geq y_{\max}|x\right)=\sum_{y\geq y_{\max}}^{\infty }\text{p}(y|x)$$

(Where N1and N2 represent the total number of reads in PA2 and PA2H (or PA4 and PA4H; or PA2H and PA4H), respectively; x and y represent the number of reads surveyed in PA2 and PA2H (or PA4 and PA4H; or PA2H and PA4H), respectively; C and D can be regarded as the probability discrete distribution of the *P*-value inspection.)

### Construction and analysis of degradome libraries

Four plant samples (PA2, PA2H, PA4 and PA4H) were used to isolate total RNA with Trizol reagent (Invitrogen) according to the manufacturer’s instructions. These samples were collected at the same time as those used for miRNAs sequencing. Poly (A) RNA was isolated from 200 μg of total RNA using the Oligotex mRNA mini kit (Qiagen). Four degradome libraries were constructed as described previously [48, 49]. Briefly, polyadenylated transcripts possessing 5′-monophosphates were ligated to the RNA adaptor containing an *Mme*I recognition site using T4 DNA ligase. Subsequently, first-strand cDNA was produced and amplified with 6 PCR cycles. Thereafter, the PCR products were purified and digested with *Mme*I, then ligated to a 3′ adaptor. Finally, the ligated products were amplified with 20 PCR cycles, and sequenced on an Illumina HiSeqTM 2000 system (Illumina, San Diego, CA, USA). After the low-quality sequences and adapters were removed from the raw reads, the remaining clean reads were analyzed by using the PairFinder software (version 2.0) [50]. Alignments with mismatch scores no more than 4 and with no mismatches at the cleave site between the 10th and 11th nucleotides were considered to be as the potential targets. These genes were classified into three categories according to the method of German et al. and Addo-Quaye et al. [48, 49]. All the identified target genes were mapped to the Nr and Nt databases, and the Swiss-Prot database by using BLASTX (E-value < 10−5) searches to obtain the target annotation. Then, Gene Ontology (GO) annotations (http://www.geneontology.org/) were conducted with the threshold of *p*-value of ≤ 0.05 [51]. Additionally, Kyoto Encyclopedia of Genes and Genomes (KEGG) pathways (http://www.genome.jp/kegg/) were also used to classify the target genes using Blastall with an E-value cutoff of 10^−5^.

### Verification of miRNAs and their potential targets by quantitative real-time PCR (qPCR)

RNA was extracted from the leaves of the PA2, PA2H, PA4, and PA4H plants using Trizol reagent (Invitrogen). The specific stem-loop primers and forward primers for the miRNAs were designed based on the mature miRNA sequences [52], and the primers for their target mRNAs were designed using Beacon Designer, version 7.7 (Premier Biosoft International, Ltd., Palo Alto, CA, USA). The U6 snRNA and 18S rRNA of *Paulownia* were used as the reference genes for the miRNAs and targets, respectively. All the primers used for the qPCR are listed in S1 Table. qPCR was carried out using a SuperScript III Platinum SYBR Green One-step qRT-PCR kit (Invitrogen) and a CFX96 Real Time PCR system (Bio-Rad, Hercules, CA, USA). The following cycles were performed: 50°C for 3 min, 95°C for 5 min, then 40 cycles, and 40°C for 10 min. Three biological and technical replicates were run for each sample. Relative expression levels were calculated using the method of Livak and Schmittgen [53].

## Results

### Deep sequencing of sRNAs in the two Paulownia genotypes

**Fig 1:**
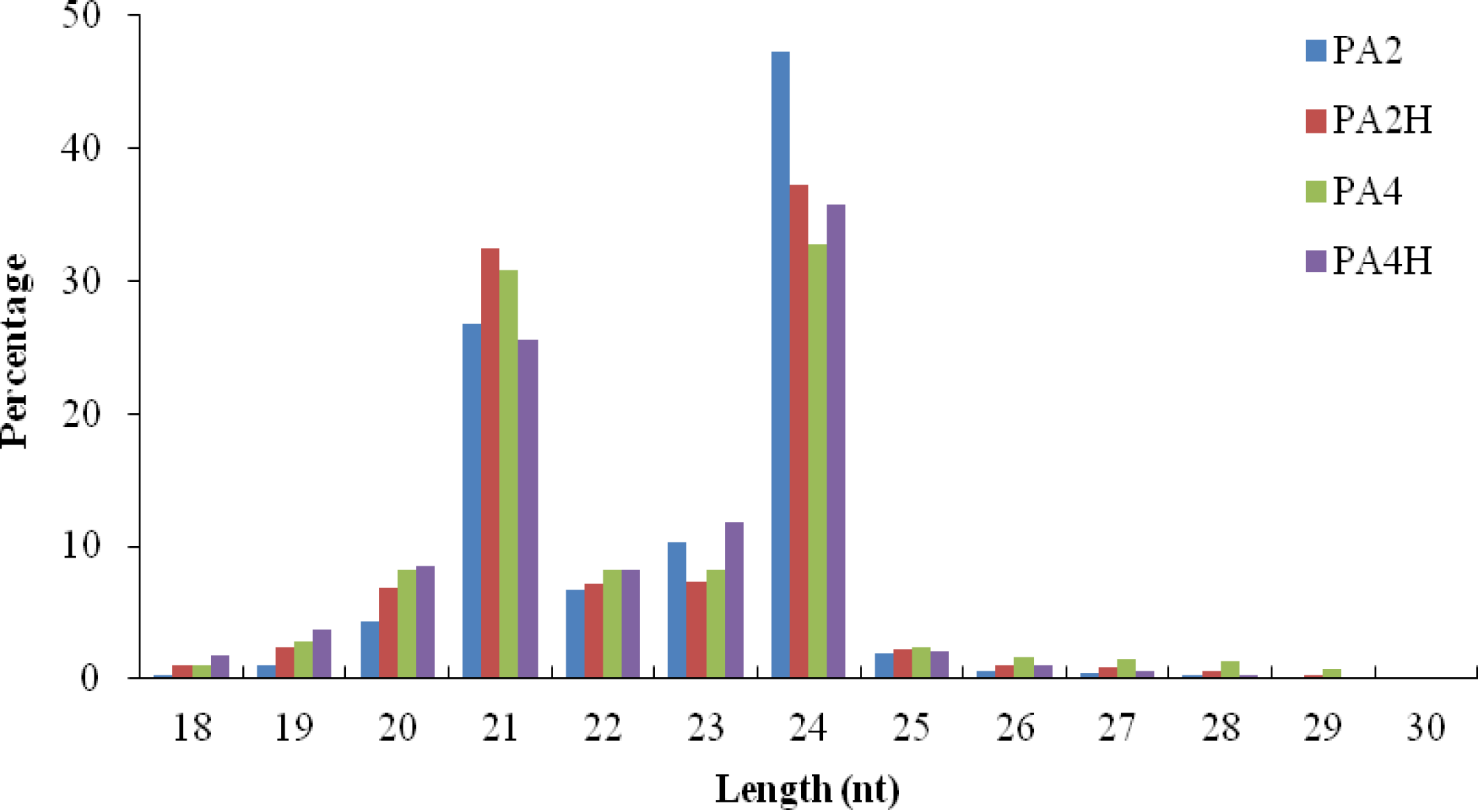
Length distribution of small RNAs obtained by Illumina sequencing in PA2, PA2H, PA4 and PA4H libraries.

A total of 14,805,381 (PA2) and 15,702,676 (PA2H) raw reads were obtained from diploid *P*. *australis*, and 15,404,659 (PA4) and 18,549,971 (PA4H) raw reads were obtained from autotetraploid *P*. *australis*. After removing low-quality reads, poly(A) reads, oversized insertions, reads shorter than 18 nt, and adaptor contaminated reads, 14,733,335 (PA2), 15,506,383 (PA2H), 15,280,087 (PA4), and 18,331,641 (PA4H) clean reads were obtained, of which 4,270,792 (PA2), 3,930,801 (PA2H), 3,691,101 (PA4), and 4,814,893 (PA4H) were unique reads (Table 1). The unique sRNAs in common between the control and drought-stressed plants were 15.78% (1,117,707 in PA2 and PA2H) and 10.53% (810,204 in PA4 and PA4H). The most abundant sequences were 24 nt in length, followed by 21 nt (Fig 1). All the clean reads that aligned to sequences in the *Paulownia* UniGenes Database (SRP031770) were classified as miRNAs, rRNAs, snRNAs, and snoRNAs based on searches against sequences in GenBank (http://www.ncbi.nih.gov/Genbank), Rfam (http://rfam.sanger.ac.uk/), and miRbase 21.0 (http://microrna.sanger.ac.uk/sequences).

**Table 1:** Annotation of sRNAs sequences from four libraries.

category	PA2				PA2H				PA4				PA4H			
	Unique sRNAs	Percent%	Total sRNAs	Percent%	Unique sRNAs	Percent%	Total sRNAs	Percent%	Unique sRNAs	Percent%	Total sRNAs	Percent%	Unique sRNAs	Percent%	Total sRNAs	Percent%
Total	4270792	100%	14733335	100%	3930801	100%	15506383	100%	3691101	100%	15280087	100%	4814893	100%	18331641	100%
miRNA	16088	0.38%	2821727	19.15%	19871	0.51%	3977020	25.65%	18497	0.50%	3062256	20.04%	23522	0.49%	2373591	12.95%
rRNA	46205	1.08%	457104	3.10%	60699	1.54%	787545	5.08%	72532	1.97%	1192127	7.80%	78618	1.63%	1345407	7.34%
snRNA	1625	0.04%	3938	0.03%	1753	0.04%	5906	0.04%	1765	0.05%	3784	0.02%	2088	0.04%	4388	0.02%
snoRNA	597	0.01%	1482	0.01%	754	0.02%	1518	0.01%	580	0.02%	1131	0.01%	775	0.02%	1604	0.01%
tRNA	8310	0.19%	455610	3.09%	14598	0.37%	757135	4.88%	16917	0.46%	893294	5.85%	13870	0.29%	1084590	5.92%
unann	4197967	98.29%	10993474	74.62%	3833126	97.52%	9977259	64.34%	3580810	97.01%	10127495	66.28%	4696020	97.53%	13522061	73.76%

### Identification of conserved and novel miRNAs in the two Paulownia genotypes

To identify the conserved miRNAs in the diploid and autotetraploid *P*. *australis* libraries, the unique reads were mapped to the known miRNAs in miRBase 21.0, allowing two mismatches. A total of 33 conserved miRNAs belonging to 16 miRNA families were identified in the four libraries (S2 Table). Among them, miR167 and miR156 were the largest families with five members each, followed by the miR398 family with four members. The 25 of them were 21 nt in length, while the remaining ones varied from 21–23 nt. Interestingly, some of the conserved miRNAs were expressed in only one of the genotypes; for example, pas-miR897 was expressed only in the diploids, and pas-miR5239 was expressed only in the autotetraploids. In addition, miRNA*s have been considered to be bona fide miRNAs, because they were found to participate in negative gene regulation using the same mechanism as miRNAs [54]. In this study, the miRNA* sequences, pas-miR156a/b/d*, pas-miR166a/b*, pas-miR168a/b*, and pas-miR396*, were found to have a relative moderate expression compared with the corresponding conserved miRNAs (S2 Table). The similar phenomenon has also been reported in *Populus tomentosa* [55]

To predict candidate novel *P*. *australis* miRNAs, the remaining unannotated unique sequences that found no matches in miRBase 21.0 were analyzed using the Mireap and RNAfold software. A total of 104 novel miRNAs with the processing precision value > 0.1 were predicted and 33 of them had corresponding miRNA*s (S3 and S4 Tables). The minimal folding free energies for the novel pre-miRNA sequences varied from −115.1 to −18.9 kcal mol^−1^, with an average of −50.74 kcal mol^−1^. The AMFE and MFEI for the each miRNAs were also calculated, and the detailed information about the pre-miRNA sequences was given in S3 Table. Eight (PA2), 17 (PA2H), 13 (PA4), and 14 (PA4H) novel miRNAs were expressed only in one of the *P*. *australis* libraries. This finding might indicate that the drought treatments generated or inhibited some novel miRNAs, which might play important roles in the drought stress response.

### Differentially expressed miRNAs in the *Paulownia australis* under drought stress

The differentially expressed miRNAs in diploid and autotetraploid *P*. *australis* under drought stress were compared as described previously [47, 56]. MiRNAs with fold changes > 1 or < −1 and with *P* ≤ 0.05 were considered to be up-regulated or down-regulated, respectively, in response to drought stress. Among the 137 (33 conserved and 104 novel) miRNAs, 54 miRNAs (15 conserved and 39 novel) were differentially expressed in diploid under drought stress (PA2H vs. PA2), while 68 miRNAs were differentially expressed in autotetraploid (PA4H vs. PA4). When comparing the impact of drought between the two ploidy levels, we found that 80 miRNAs (16 conserved and 64 novel) were differentially expressed in the PA2H relative to PA4H (S2 and S3 Tables). Interestingly, we found only 21 miRNAs (12 conserved and 9 novel) that were both differentially expressed in two *Paulownia* genotypes.

### Target identification for the two Paulownia genotypes by degradome analysis

**Fig 2:**
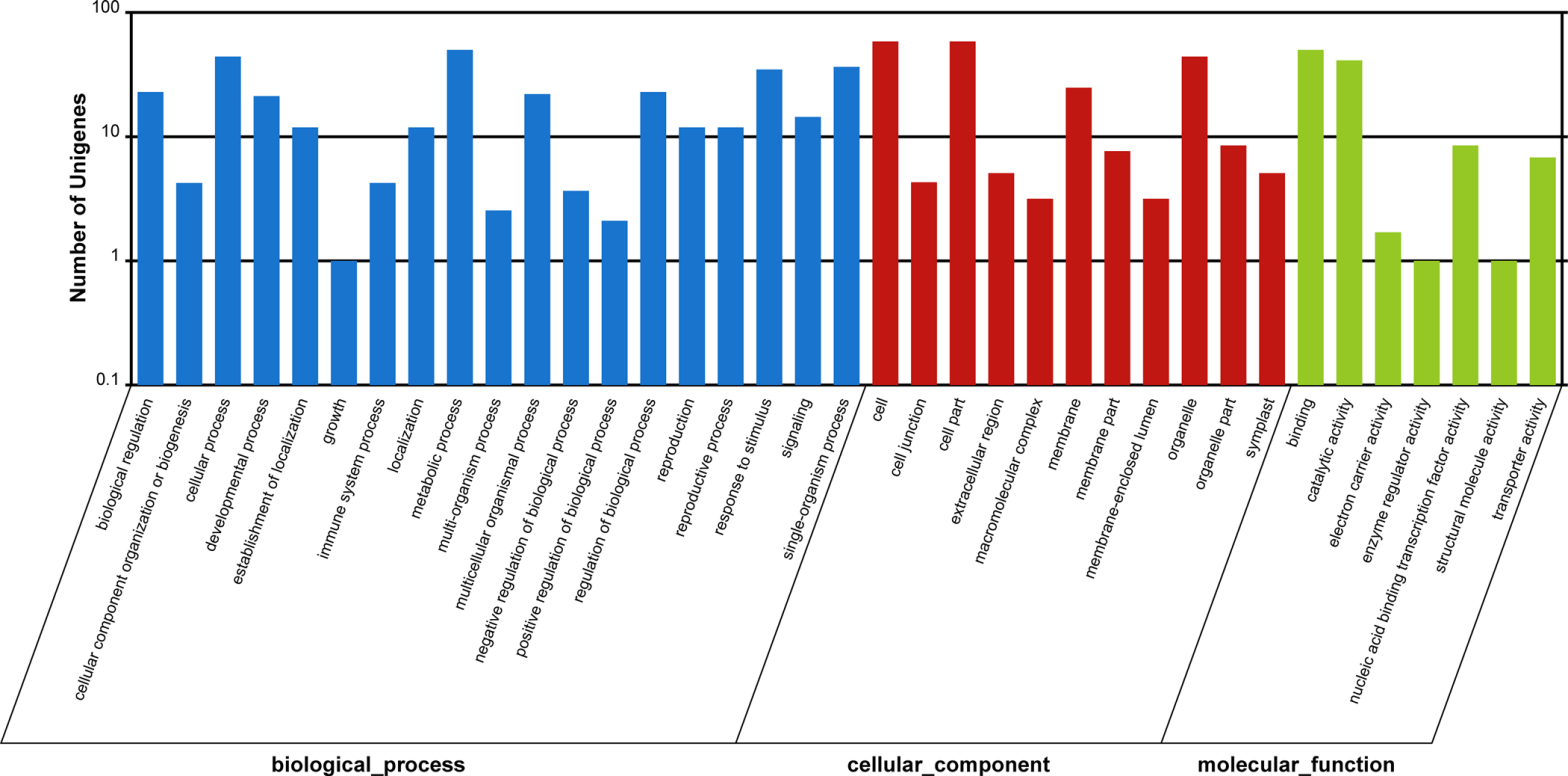
Gene Ontology analyses of the miRNAs targets in *P*. *australis*.

We identified 125 genes from four degradome libraries targeted by 36 miRNAs. All the targets were pooled and classified into three categories based on their relative abundances [48, 49] (114 targets (121 cleavage sites) were assigned to category І, 39 targets (66 cleavage sites) were assigned to category II, and 10 targets (21 cleavage sites) were assigned to category III) (S5 Table). The target genes were mapped to known genes in the GenBank Nr and Nt databases, and in the Swiss-Prot database by BLASTX searches and annotated based on the annotations of the corresponding homologous sequences. The majority of annotated target genes encoded squamosa promoter-binding-like proteins, auxin response factors, serine/threonine-protein kinase AtPK2/AtPK19, putative hypersensitive-induced response proteins, putative disease resistance proteins, E3 ubiquitin-protein ligase RGLG2-like, ABC transporter C family members, nuclear transcription factor, WRKY transcription factor, and predicted proteins (S5 Table). Moreover, a GO analysis of the assigned functional annotations under the biological process, cellular component, and molecular function categories was performed (a *p*-value of ≤ 0.05 was used as the threshold) as described previously (Fig 2) [51]. The target genes were classified further using the KEGG Pathway database, which showed that the target genes were predicted to be involved in 89 pathways, including mainly plant hormone signal transduction, starch and sucrose metabolism, cyanoamino acid metabolism, and phenylpropanoid biosynthesis (S5 Table). The annotation of these miRNA target genes may provide some new insights into how *P*. *australis* miRNAs regulate gene expression under drought stress.

### Validation of miRNAs and their targets in the Paulownia by qPCR

**Fig 3:**
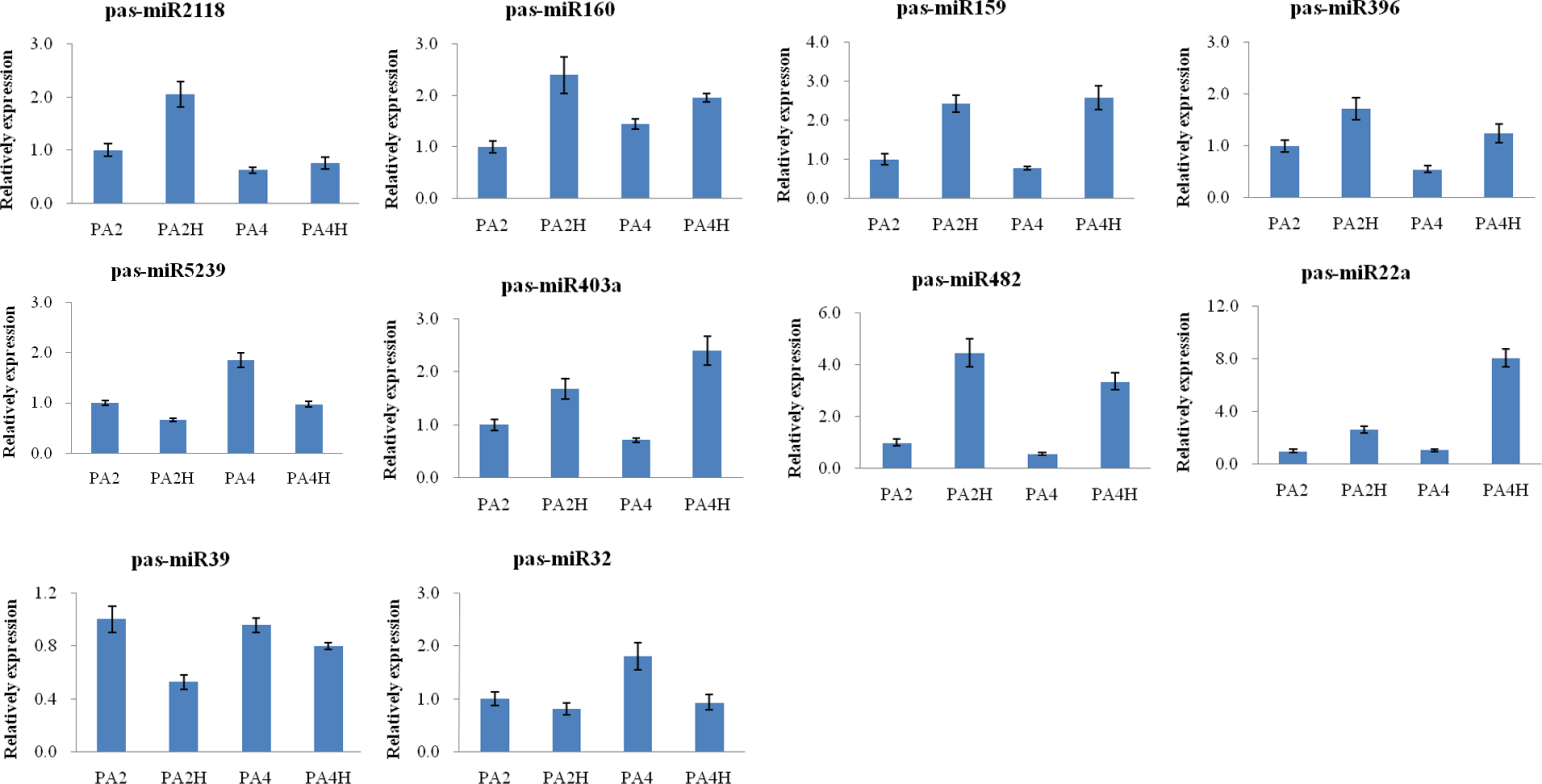
Results from qPCR of miRNAs in PA2, PA2H, PA4 and PA4H plants. Three independent biological replicates for each sample and three technical replicates of each biological replicate were performed. U6 snRNA gene was chosen as the endogenous control. The normalized miRNA levels in the PA2 were arbitrarily set to 1. The error bars were indicated on each column.

**Fig 4:**
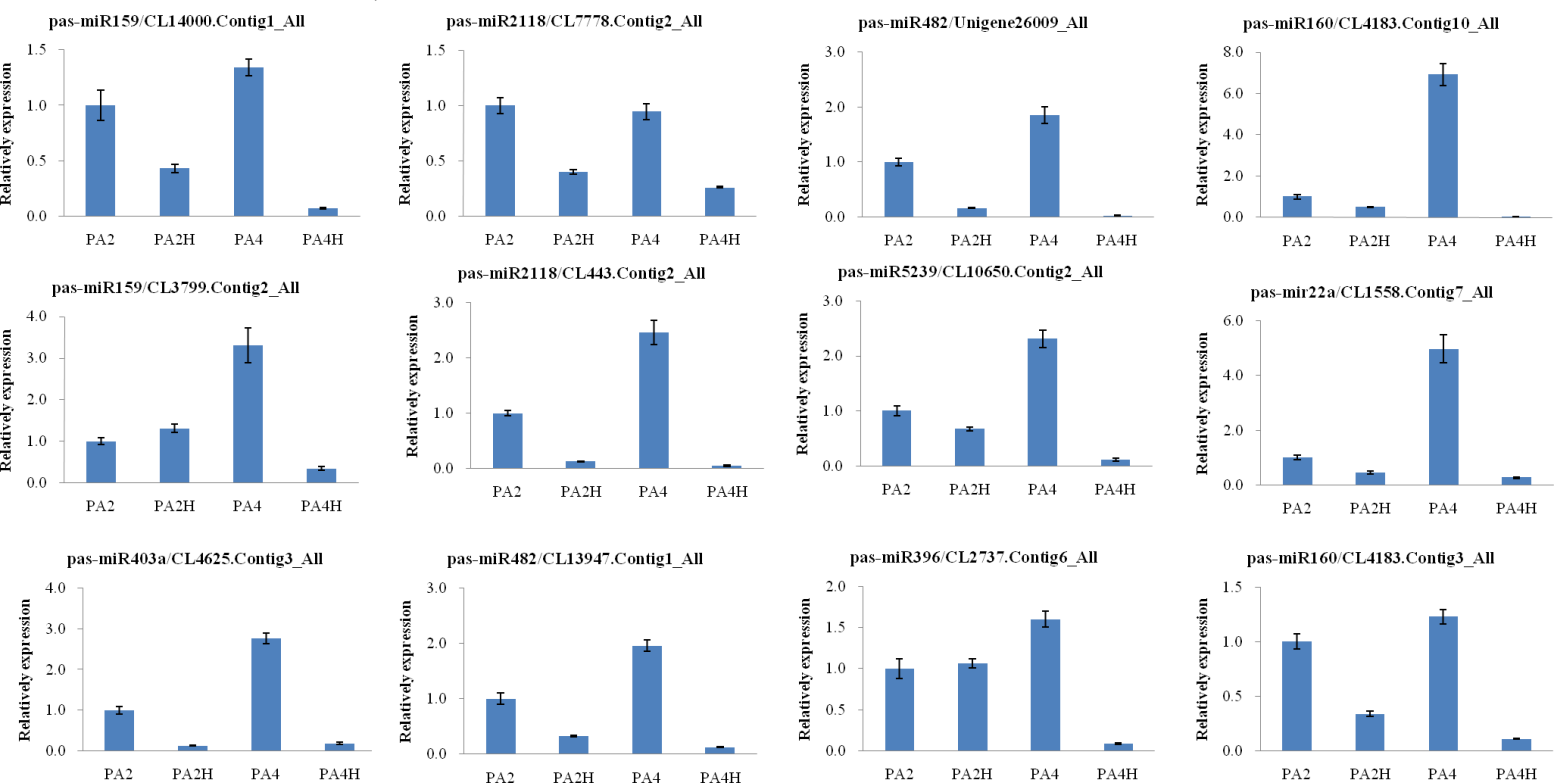
Relative expression levels of the target genes in *P*. *australis*. Three independent biological replicates for each sample and three technical replicates of each biological replicate were performed. 18SrRNA was chosen as the endogenous control. The normalized miRNA levels in the PA were arbitrarily set to 1. The error bars were indicated on each column. CL14000.Contig1_All and CL3799.Contig2_All targeted by pas-miR156; CL7778.Contig2_All and CL443.Contig2_All targeted by pas-miR2118; Unigene26009_All and CL13947.Contig1_All targeted by pas-miR482; CL4625.Contig3_All targeted by pas-miR403a; CL10650.Contig2_All targeted by pas-miR5239; CL2737.Contig6_All targeted by pas-miR396; CL4183.Contig10_All and CL4183.Contig3_All targeted by pas-miR160; CL1558.Contig7_All targeted by pas-miR22a.

To verify the miRNA expression levels predicted from the sequencing data and the potential correlation between the miRNAs and their predicted targets, 10 of the differentially expressed miRNAs and 12 of their target genes were selected randomly for the qPCR array. As shown in Fig 3, the expression patterns of the 10 selected miRNAs showed similar trends in the qPCR analyses and the sequencing data; seven were up-regulated and three were down-regulated in the drought-stress plants compared with the controls. Furthermore, 12 target genes for the eight miRNAs also were examined by qPCR. The results showed that the expression patterns of 11 of the target genes (CL14000.Contig1_All, CL7778.Contig2_All, Unigene26009_All, CL4183.Contig10_All, CL3799.Contig2_All, CL443.Contig2_All, CL10650.Contig2_All, CL4625.Contig3_All, CL13947.Contig1_All, CL2737.Contig6_All, and CL4183.Contig3_All) were negatively correlated with the expression patterns of their corresponding miRNAs (Fig 4). The expression level of only one of the 12 target genes, CL1558.Contig7_All, showed the same trend as the corresponding miRNA pas-miR22a. These findings indicated that the Illumina sequencing analysis was reliable, and also provided further experimental verification of the response of these miRNAs to the drought stress in the diploid and autotetraploid *P*. *australis* plants.

## Discussion

### Drought responsive miRNAs in *P*. *australis*

MiRNAs play important regulation roles in plant growth, development, and biotic and abiotic stress response [19, 20, 22–24, 57–63]. Since the first Paulownia miRNA was discovered in *Paulownia tomentosa* [23], a large number of miRNAs have been identified from different Paulownia species by using the Illumina sequencing platform [37–40]. *P*. *australis* is regarded as an ideal species to study the mechanisms of drought tolerance because of its fast growth and inherent ability to resist the extreme environmental conditions [64]. Previous studies have shown that the changes in some of physiological and biochemical indexes have the similar trends both in the diploids and autotetraploids under the water deficit [9]. In this present study, therefore, we constructed four sRNA libraries and four degradome sequencing libraries from well-watered and drought-treated diploid and autotetraploid *P*. *australis* plants to discover the miRNAs and their target genes that might be associated with the drought stress in *P*. *australis* plants.

We previously detected 45 conserved and 31 novel miRNAs in the diploid and autotetraploid *P*. *australis* [39]. In the present study, we detected 33 conserved miRNAs belonging to 16 miRNA families and 104 novel miRNA candidates from the four sRNA libraries. This obvious fewer conserved miRNAs and more novel miRNAs in the present study than in the our previous study [39] is likely because the previous study was between the two ploidy level under non-stressed condition, but the plants in the present study had drought stress. This means that some differential expressed novel miRNAs were associated with drought stress. Some miRNAs of the MIR164 family were previously detected in Niu et al. [39] but not been detected in this study, whereas three miRNA families including MIR403, MIR5239 and MIR897 were discovered in the *P*. *australis* plants for the first time (S2 Table) [39]. A similar phenomenon has also been presented in the identification of cotton miRNAs [65, 66]. In addition, different reference transcriptome databases have been used to identify the potential miRNAs between Niu et al. [39] and the present study. Moreover, here, the MFEI value and the processing precision rates were applied to validate their authenticity in the present study. Additionally, a total of 101 differentially expressed miRNAs were detected in the plants under drought stress; 54 and 68 were identified in diploids and autotetraploids, respectively, and 21 were found to be common in diploids and autotetraploids. Some of the differentially expressed miRNAs (pas-miR171a/b, pas-mir15, pas-mir16, pas-mir34, pas-mir39, pas-mir60, pas-mir68, and pas-mir84) were increased or decreased more than 5 folds under the drought treatment. Specifically, pas-mir84 is the most decreased miRNA (-10.51 fold in diploids, and -9.83 fold in the autotetraploids) and pas-mir68 is the most increased miRNA (9.24 folds in diploids, and 8.62 fold in the autotetraploids) in response to drought stress. The identification of these drought-stress responsive miRNAs in *Paulownia* may allow a better understanding of the miRNAs involved in defense responses, potentially leading to breeding improved drought-resistant trees.

### Drought responsive targets for *P*. *australis* miRNAs

The degradome analysis identified 125 genes that were targeted by 36 miRNAs, some of which were differentially expressed in drought-stressed *P*. *australis* plants. Many of the target genes were predicted to encode proteins involved in DNA binding, siRNA binding, protein binding, calcium lipid binding, metal ion binding, zinc ion binding, protein serine/threonine kinase activity, and response to stress. Different miRNA family members are known to have different functions in plant growth. It has been reported that members of the miR156 family were significantly repressed in maize under drought stress, and their target genes have been predicted to be involved in regulating diverse physiological processes such as, evolution of maize from teosinte, flowering time, affects on sterility, and response to salt stress [24, 29, 67]. In our study, pas-miR156c and pas-miR157 were significantly expressed in the drought-treated plants, while the expressions of most members of the miRNA156 family were not altered under drought stress. The target genes of pas-miR156c and pas-miR157 were predicted to encode the squamosa promoter-binding-like proteins SPB12, SPB16, and SPB4, which were transcription factors reported to be stress responsive in *Arabidopsis*, rice, and maize [24, 29, 68]. We also found that pas-miR156c was significantly up-regulated in the drought-treated diploids, while pas-miR157 was significantly down-regulated in the drought-treated autotetraploids. This result is similar to previous findings that the expression of miR156 increased significantly in *Arabidopsis* dehydration stress groups when compared with the control group [68]; In *Populus euphratica*, miR156 was first up-regulated then down-regulated later [69]; however, in *Populus tomentosa*, miR156 was down-regulated in a drought-treated library [55]. These findings indicated that the stresses-induced miRNA expression had dynamic variation among treatment stages. The miRNAs, pas-miR159 pas-miR160, pas-miR403a/b, and pas-miR2118 formed another group of the drought responsive miRNAs that were upregulated in both the PA2H and PA4H libraries. They were predicted to target the GAMYB transcription factors, auxin response factors, protein argonaute 2 (AGO2A2), and putative late blight resistance proteins, respectively. MYB transcription factors have been shown to play regulatory roles in essential processes of growth and development (such as flowering time and anther development leaf) and defense responses in plants [70]. In *Arabidopsis*, over-expression of Myb genes has been reported to improve tolerance to freezing, drought, and salt stresses [71]. Auxin response factors belong to a class of transcription factors that regulate multiple processes in plants, including regulation of gynoecium and stamen maturation, seed dispersal, and responses to environmental stress [58, 72], suggesting that pas-miR160 may be involved in stress-resistance in *P*. *australis* by influencing the auxin signaling pathways. AGO2 and putative late blight resistance proteins are encoded by two plant-pathogen-related genes. The putative late blight resistance proteins can guard plants against pathogens that contain an appropriate avirulence protein via an indirect interaction with the avirulence protein [73]. This interaction can trigger a plant’s defense system, including the hypersensitive response, and restrict pathogen growth [73]. In a previous study, it was reported that AGO2 performed its potential roles in plant immunity by regulating the expression of many genes through binding to a set of functional sRNAs. These findings suggested that pas-miR403a/b and pas-miR2118 may be responsive to multiple stresses, and may play important roles in *P*. *australis* plants under drought or dehydration stress.

### Targets for the candidate novel miRNAs are involved in response to drought stress

Many target genes of the novel miRNAs identified in this study were predicted to have a role in plant response to drought or water scarcity stresses. For example, the target genes of pas-miR79 were predicted to encode a stress-induced zinc-finger protein, alcohol dehydrogenase, and pentatricopeptide repeat-containing protein. Zinc finger CCCH domain-containing protein is a RNA-binding protein and many studies have shown that it may be regulated by abiotic or biotic stresses, and could have regulatory functions in mRNA processing [74–77]. Alcohol dehydrogenase has been reported to respond to oxidative stress and cadmium ions according to the gene ontology annotation. Pentatricopeptide repeat proteins share certain features with disease resistance genes, and it has been suggested that their “nomadic” character may indicate that their evolutionary expansion in plants involved novel molecular processes and selective pressures [78]. Hence, pas-miR79 may play a critical role in drought stress resistance in *Paulownia*. We also found that the ABC transporter C family member 3-like gene family targeted by pas-miR22 encodes transmembrane proteins related to the transport of indole-3-acetic acid [79, 80], indicating that pas-miR22 might affect growth and development in the *Paulownia* under drought stress. Three candidate novel miRNAs (pas-miR32, pas-miR41, and pas-miR66) were identified to target transcription factors, including WRKY transcription factor 2, nuclear transcription factor Y subunit A-3 (NF-YA3), and NF-YA4, that have functions associated with development and meristem maintenance or identity, defense/stress signaling pathways, and modulators of the expressions of other genes that respond to dehydration stress [81–83]. Unfortunately, we did not find the target sequences of some of novel miRNAs including pas-mir15, pas-mir16, pas-mir34, pas-mir39, pas-mir43, pas-mir60, pas-mir68, and pas-mir84 although these miRNAs were induced or suppressed by the water deficit in the present study. Further studies will focus on the biological functions of novel miRNA-mediated target genes in response to the drought stress in *P*. *australis* when the reference Paulownia genome has been published.

In this study we used large-scale transcriptome data to identify and analyze conserved and novel miRNAs in diploid and autotetraploid *P*. *australis* under drought stress. We identified 33 conserved and 104 novel miRNAs, 21 of which were differentially regulated in both *P*. *australis* genotypes under water scarcity stress. Bioinformatics analysis of the miRNA expression patterns and the biological functions of their targets showed that these miRNAs and target genes were involved in complex drought stress response pathways. These findings provide a basis for future investigations of miRNAs that respond to drought stress in *Paulownia* plants, and may help elucidate the molecular mechanisms that underlie environmental adaptations in *P*. *australis*.

## Supporting Information

S1 Table qRT-PCR validated miRNAs and their target genes primers. (XLSX)

S2 Table The conserved *P*. *australis* miRNAs identified from four libraries. (XLSX)

S3 Table The Novel miRNAs identified from four libraries. (XLSX)

S4 Table The processing precision values for the potential novel miRNAs. (TXT)

S5 Table Identified targets of miRNA involved in *P*. *australis* by degradome analysis. (XLSX)
